# Raspberry leaf (Rubus idaeus) use in pregnancy: a prospective observational study

**DOI:** 10.1186/s12906-024-04465-7

**Published:** 2024-04-22

**Authors:** Rebekah L. Bowman, Jan Taylor, Deborah L. Davis

**Affiliations:** 1 University of Canberra, Canberra, Australia; 2 University of Canberra and ACT Government, Health Directorate, Canberra, Australia

**Keywords:** Pregnant women, Pregnancy, Complementary Therapies, Complementary and Alternative Therapies, Raspberry leaf

## Abstract

**Background:**

Raspberry leaf use during pregnancy in Australia is widespread. There has been little research exploring the potential beneficial or harmful effects of raspberry leaf on pregnancy, labour, and birth. More research is needed to appropriately inform childbearing women and maternity healthcare professionals on the effects of raspberry leaf so that women can make informed choices.

**Methods:**

This study aimed to determine associations between raspberry leaf use in pregnancy and augmentation of labour and other secondary outcomes. Data was derived from questionnaires which captured demographic information and herbal use in pregnancy. Clinical outcomes were accessed from the maternity services’ clinical database. Data analysis was conducted in R via package ‘brms’ an implementation for Bayesian regression models.

**Results:**

A total of 91 completed records were obtained, 44 exposed to raspberry leaf and 47, not exposed. A smaller proportion of women in the raspberry leaf cohort had augmentation of labour, epidural anaesthesia, instrumental births, caesarean section, and postpartum haemorrhage. A larger proportion had vaginal birth and length of all phases of labour were shorter. Under these conditions the use of raspberry leaf was strongly predictive of women not having their labours medically augmented.

**Conclusions:**

While our study demonstrated that raspberry leaf was strongly predictive of women not having their labours medically augmented, the results cannot be relied on or generalised to the wider population of pregnant women. While there were no safety concerns observed in our study, this should not be taken as evidence that raspberry leaf is safe. A randomised controlled trial is urgently needed to provide women and healthcare providers with robust evidence on which to base practice.

**Supplementary Information:**

The online version contains supplementary material available at 10.1186/s12906-024-04465-7.

## Background

Raspberry leaf use during pregnancy in Australia is widespread [1]. While childbearing women and midwives in Europe have been using the herb for more than a century, there is scant evidence to inform contemporary practice (2). Historically raspberry leaf, *Rubus idaeus* of the Rosaceae family, (2) has been used to strengthen and tone the uterus, theoretically assisting contractions and checking any haemorrhage [2, 3]. Strengthening the tone of the uterus may help women avoid intervention in labour and birth including for example augmentation of labour. Augmentation of labour is recommended for prolonged spontaneous labour. Augmentation involves stimulating the uterus to increase frequency, duration and intensity of contractions [4]. Augmentation of labour is a common intervention in Australia. In 2022, 39% of all Australian women having their first baby had their labours augmented [5].

Raspberry leaf is one of the most common herbs used in pregnancy. Recently, researchers surveyed 121 women who had given birth in Queensland with the aim to identify how common the use and knowledge of raspberry leaf was during pregnancy. Of these, 73% were aware of the practice with 38% reporting using raspberry leaf in their pregnancy [6]. A larger survey of 810 Australian women who used herbal medicine during their pregnancy, found raspberry leaf to be the most common herb used, with ginger a close second [7].

Midwives also frequently recommend raspberry leaf to childbearing women. Over half of the midwives surveyed in Australia in 2017 recommended raspberry leaf to women experiencing a post-dates pregnancy. It was also the most common complementary therapy that midwives used in their own pregnancies [8].

While there is a long history of empirical use, there has been little research on the benefits or potential harmful effects of raspberry leaf on pregnancy, labour and birth. An integrative review of animal and human studies demonstrated contradictory effects of raspberry leaf in In Vitro and In Vivo studies, on animal and human uteri and smooth muscle [9].

In laboratory and animal studies raspberry leaf was shown to contain active constituents that demonstrated both stimulatory and relaxation effects on smooth muscle depending on factors such as preparation, method of extraction, and type of tissue. These studies described numerous phytochemicals contained in raspberry leaf including tannins, phenolic acids, terpenoids ad flavonoids [10–15]. In human studies, three retrospective observational studies have examined the association between raspberry leaf and maternity outcomes. Bohata and Dostalek [16] focused on perineal outcomes finding no statistically significant association between raspberry leaf and perineal outcome. A small sub sample of women (*n* = 34) in a study focusing on herbal remedies more broadly [17], identified an association between raspberry leaf use and increased rate of caesarean Sect. (23.5% vs. 9.1%; adjusted OR 3.47; 95% CI 1.45–8.28). The small sample size, selection bias, and failure to account for confounders, limits the reliability of this finding. In a larger study, of 108 multiparous and primiparous women (57 raspberry leaf and 51 control) Parsons, Simpson and Ponton [18], examined associations between raspberry leaf consumption and augmentation of labour, length of labour and with several neonatal outcomes. There were no statistically significant differences in any outcomes including rates of augmentation of labour or length of labour though the length of labour for the raspberry leaf group was shorter than the control group in the second and third stages of labour by 48 and 6 min respectively. This is a clinically meaningful finding though the small sample size and lack of control of confounding variables such as parity, similarly, limits the reliability of these findings.

A follow-up randomised, double-blind, placebo-controlled trial was conducted by the same research team involving 192 women from 32 weeks of pregnancy to labour, this time limiting the cohort to primiparous women. The women treated were given 1.2 g of raspberry leaf twice daily and the outcomes of interest included induction of labour, augmentation of labour, length of labour, and mode of birth with average length of labour the primary outcome. After excluding women who had labours induced, only 70 were included in analyses examining augmentation of labour. No adverse outcomes were reported, and no statistically significant differences were found between groups. Women in the raspberry leaf group had a shorter second stage of labour (by 9.6 min), which is clinically meaningful.

The researchers used a conservative dose of 2.4 g /day from 32 weeks gestation which was required for ethical approval [19]. This is significantly below the recommended dose of the British Herbal Medicine Association (4–8 g) [20]. The popular Australian company “Herbs of Gold” sells raspberry leaf tablets to pregnant women over the counter in pharmacies and health food shops, or online. Their recommended dose is 4 g per day – nearly double what was used in the research [21].

More research is needed to appropriately inform childbearing women and maternity healthcare professionals on the effects of raspberry leaf on pregnancy, labour, and birth so that women can make better informed choices. This study aims to build on previous research with appropriate control of confounding variables to determine associations between raspberry leaf use in pregnancy, augmentation of labour and other selected outcomes.

## Methods

While a randomised controlled trial would provide the strongest evidence, preliminary discussions with our Ethics Committee suggested that such as study would be treated as a drug trial and require phase 1 and 11 testing. This was beyond our resources and therefore we decided to proceed with a prospective observational study, addressing some of the limitations of previous studies.

The purpose of this prospective observational study was to determine associations between raspberry leaf use in pregnancy and augmentation of labour and other secondary outcomes. The study was conducted in one jurisdiction in Australia with two maternity facilities: one providing care to approximately 1,500 women per annum and the other 3,500. Women planning to birth in either of these facilities were eligible to participate. Other inclusion criteria included age 18–40, primiparous with singleton pregnancy, not smoking cigarettes, vaping, or using non prescribed drugs or alcohol, BMI 18.5–35 with no major co-morbidities, and able to communicate in English. Women were excluded if they developed complications such as gestational diabetes or hypertension and if they experienced induction of labour.

Promotion flyers were prepared for the study and distributed via antenatal clinics, childbirth preparation classes and social media. The study was titled “herbal use in pregnancy” and highlighted that women who were and were not using herbal remedies in pregnancy were eligible to participate. Flyers contained a QR code that linked directly to participant information and consent process. After reviewing the information all participants provided consent before continuing with the online questionnaire. A follow up questionnaire was sent to all participants every 2 weeks to capture those commencing raspberry leaf after the last completed questionnaire. The questionnaire and follow up questionnaires are available as a supplementary file.

Recruitment for this study was impacted by the COVID-19 pandemic with recruitment beginning July 2019 and ending November 2022. Data derived from a series of questionnaires which captured demographic information (first questionnaire) and herbal use in pregnancy was collected every two weeks from 30 weeks gestation. Those that used raspberry leaf in any form or dose at any gestation formed the “raspberry leaf” cohort and those that did not use any raspberry leaf during pregnancy, the “non raspberry leaf” cohort. Clinical outcomes were accessed from the maternity services’ clinical database including augmentation of labour, gestation, analgesia used in labour, mode of birth, length of labour (1st, 2nd and 3rd stages), measured blood loss, postpartum haemorrhage (PPH) > 500 ml and > 1000 ml, iron transfusion, neonatal Apgar score, and admission to neonatal nursery (NICU). For the purposes of this study, augmentation of labour is defined as the intravenous administration of synthetic oxytocin (with or without artificial rupture of the membranes) to augment a labour that has commenced spontaneously. Ethical approval for the study was obtained from the relevant Human Research Ethics Committees of each facility involved (REGIS: 2018/ETH00271; ACT Health: 2018/LRE/00125; Calvary: 30-2108).

### Statistical analysis

Data analysis was conducted in R [22] via package ‘brms’ [23] an implementation for Bayesian regression models. A Bayesian approach using weakly informative priors was selected to provide regularization, necessary for categorical outcomes with a small sample size [24]. The binomial response variables were assessed using generalized linear models with binomial response distribution and logit link, with weakly informative priors for the coefficients. Effects of the predictors were summarized as the odds ratio. Birth mode (vaginal, assisted, caesarean) was described using a multinomial model with logit link. The effect of raspberry leaf exposure on birth mode was expressed as the conditional odds. All models included a p-spline [25] to control for age. Predictions were expressed for the sample median age (30y).

Using a confidence level of 95%, with 80% power, presuming a ratio of 1:1 and a two tailed test, a sample size of 756 women was needed to demonstrate a reduction of augmentation from 28.9% (augmentation rate at the time of commencing the study) [5] to 20%. After several hundred women were recruited, only 91 met the selection criteria described above. The decision to continue to recruit was re-evaluated as recruitment to the selection criteria was very slow and there were significant issues recruiting due to the COVID-19 pandemic. A statistician was consulted, and it was apparent the effect on the primary outcome (augmentation) was overwhelmingly large (see Table 1). It was determined that the collection of more data was not likely to influence the results and therefore a decision was made to cease recruitment. Consequently, the decision to continue with the study despite the small sample size was made.

**Table 1:** Participant characteristics

Characteristics	Raspberry leaf *n* = 44 M(SD) or n (%)	Non- Raspberry leaf *n* = 47 M(SD) or n (%)
Mean Age	30.25 (3.6)	29.4 (2.98)
INCOME		
Income < $80K	7 (16%)	4 (9%)
Income > $80K	37 (84%)	43 (91%)
EDUCATION		
High School	6 (14%)	7 (14%)
Trade / Diploma	2 (5%)	5 (11%)
Tertiary	36 (81%)	35 (75%)
MARITAL STATUS		
Defacto/Married	43 (98%)	47 (100%)
Single	1 (2%)	0
MODEL OF CARE		
Continuity of midwifery care	34 (77%)	24 (51%)
Standard model of care	10 (23%)	23 (49%)

## Results

### Descriptive results

A total of 91 completed records were obtained, 44 exposed to raspberry leaf and 47, not exposed. Table 2 shows the characteristics of participants. Most participants were married or partnered and had tertiary level education. More women experiencing continuity of midwifery compared to standard care, were in the raspberry leaf group.

**Table 2:** Raspberry leaf exposure

Exposure	n (%) or Mean (range)
Gestation commenced	36 weeks (range 8–38 weeks)
FORM	
Tea	37 (84%)
Tablet	4 (9%)
Both tablet and tea	3 (7%)
DOSAGE	
Tea	Mean 2 cups (range 1–6 cups)
Tablet	Mean 4 mg/day (range 3–4 mg /day)
Tea and tablet consumed	4 mg day, with sporadic cup of tea

Table 3 presents descriptive data on raspberry leaf use. Of the 91 women included in analysis, 44 (48%) were exposed to raspberry leaf. The most common time women commenced raspberry leaf was at 36 weeks gestation, but some started as early as 8 weeks and some as late as 38 weeks. Women were much more likely to consume the tea (84%) than the tablet (9%). Three women consumed both the tablet and the tea. There was great discrepancy in the dosage regime of the tea, with women having a cup of raspberry tea between a range of 1 and 6 times a day. The limitation of a small sample size has restricted the analysis of association between raspberry leaf and the effect of dose on augmentation of labour.

**Table 3:** Primary and secondary outcomes

Outcome	Raspberry leaf *n* = 44 Median or n (%)	Non- Raspberry leaf *n* = 47 Median or n (%)
Augmentation of labour	1 (2%)	32 (68%)
ANALGESIA IN LABOUR		
Epidural	6 (14%)	26 (55%)
Morphine	0	2 (4%)
Nitrous Oxide	9 (20%)	11 (23%)
Gestation at birth	40.1 (38–41 + 5)	40.3 (38 + 1–42 + 2)
MODE OF BIRTH		
Vaginal	33 (75%)	25 (53%)
Instrumental	3 (7%)	17 (36%)
Caesarean section	5 (11%)	8 (17%)
LENGTH OF LABOUR		
1st stage	7:45 (1:45 − 10:18)	8:23 (2:44 − 12:10)
2nd stage	1:33 (00:17 − 2:44)	2:45 (00:19 − 4:46)
3rd stage	12:45 (0:04 − 0:26)	13:15 (0:01 − 0:26)
Measured blood loss	370 (50–800) mls	475 (300–2800) mls
PPH	6 (14%)	13 (28%)
Iron transfusion	0	0
Apgar score < 7	4 (9%)	7 (15%)
NICU admission	0	0

Table 1 shows primary and secondary outcomes. A smaller proportion of women in the raspberry leaf cohort had augmentation of labour, epidural anaesthesia, instrumental births, caesarean section, and postpartum haemorrhage (PPH). A larger proportion had vaginal birth. Measured blood loss was smaller in the raspberry leaf group and length of all phases of labour were shorter. A smaller proportion of neonates had Apgar scores of < 7 in the raspberry leaf group.

### Regression analyses

Logistic regression was used to determine the association between raspberry leaf use and augmentation of labour. Table 4 shows results of this analysis controlling for the effect of epidural, continuity of care and age.

**Table 4:** Logistic regression for augmentation of labour controlling for age

	Estimate (odds ratio)	Q5	Q95
Intercept	1.64	0.67	4.21
**Raspberry Leaf NO**	1	-	-
**Raspberry leaf YES**	0.05	0.02	0.15
**Epidural NO**	1	-	-
**Epidural YES**	5.11	1.96	13.84
**Continuity of Care NO**	1	-	-
**Continuity of Care YES**	0.56	0.20	1.50

Under these conditions the use of raspberry leaf was significantly and strongly predictive of women not having their labours medically augmented. In this model, the odds of augmentation of labour was lower (0.05 times the odds) in the presence of raspberry leaf than in its absence. This analysis also demonstrates that in the presence of epidural anaesthesia there was significantly greater odds (more than 5 times) of augmentation of labour. Model of care was not significantly associated with augmentation of labour. Some of the credible intervals are wide and reflect the small sample size and a lack of precision of the estimate.

Associations between raspberry leaf and mode of birth were examined controlling for the effect of age. A multinomial (categorical) logistic regression was used. Table 5 presents these results.

**Table 5:** Conditional odds for raspberry leaf and mode of birth (controlling for effect of age)

Mode of Birth	RL	Estimate (odds)	Q5	Q95
Vaginal	No	0.98	0.58	1.61
**Vaginal**	**Yes**	**4.48**	**2.29**	**9.42**
Assisted	No	0.56	0.32	0.95
**Assisted**	**Yes**	**0.08**	**0.03**	**0.20**
Caesarean	No	0.16	0.08	0.31
**Caesarean**	**Yes**	**0.11**	**0.04**	**0.26**

Controlling for age, raspberry leaf use was associated with significantly greater odds of vaginal birth and significantly lower odds of assisted birth and caesarean section. In the presence of raspberry leaf there were 4.5 vaginal births for every one birth of any other type. The intervals in this table are wide reflecting the small number of cases.

Postpartum haemorrhage and its association with raspberry leaf use was examined using logistic regression controlling for mode of birth, augmentation of labour and age. Results are presented in Table 6.

**Table 6:** Logistic regression for raspberry leaf and postpartum haemorrhage controlling for age

	Estimate	Q5	Q95
Intercept	0.05	0.01	0.19
Raspberry leaf	1.83	0.47	8.85
Mode of birth			
Vaginal	ref		
**Assisted**	**3.48**	**1.16**	**11.14**
Caesarean section	2.37	0.61	8.52
**Augmentation of labour**	**4.26**	**1.23**	**18.34**

There was no meaningful evidence that raspberry leaf use was associated with an increase or reduction in postpartum haemorrhage though the odds of PPH was increased in assisted modes of birth and augmentation of labour. Again, wide confidence intervals reflect the small sample size and a lack of precision of the estimate.

## Discussion

Results of this study suggest that raspberry leaf use in pregnancy is associated with a reduction in augmentation of labour, the primary outcome for this study. It is also associated with an increase in vaginal birth and no association was found with postpartum haemorrhage. These results must be interpreted with caution due to the small sample size and likely selection bias.

The study eligibility criteria meant that the sample was comprised of women at low risk of obstetric complication. This is reflected in the low overall rates of caesarean section at 14% compared to the average in the ACT which is 42.1% [5]. This, however, would have impacted both groups equally. Recruitment methods also resulted in a large proportion of women from midwifery continuity of care models (63%) which is higher than the ACT average being 38% [26]. In the raspberry leaf cohort 77% of women experienced continuity of care while the in the non-raspberry leaf group it was 51%. Women choosing continuity of midwifery care are typically more aligned to natural birth philosophies, seeking vaginal birth with low levels of intervention. The proportion of women using raspberry leaf in our study is higher than that reported in other Australian studies at 48% vs. 38% [6]. Our systematic review with meta-synthesis found that women were using complementary and alternative medicine in pregnancy as a means to support their sense of self-determination, to pursue a natural and safe childbirth, and because they experience a close affiliation with the philosophical underpinnings of complementary and alternative medicine as an alternative to the biomedical model [27]. This group may have other characteristics not measured, that make them different from the non-raspberry leaf cohort and they may be more likely to decline intervention in labour and birth such as augmentation of labour. While efforts were made to control for contributing factors (age, model of care, epidural use) other unmeasured, unidentified factors may have contributed to the results.

In our study, descriptive statistics showed that fewer women in the raspberry leaf cohort had caesarean section or assisted modes of birth (11% vs. 17% and 7% vs. 36% respectively) and shorter labours particularly second stage. They also had smaller overall blood loss. Some of these findings resonate with that of Simpson et al. [19] who found women using raspberry leaf had a clinically significant (though not statistically) shorter second stage of labour (by almost 10 min), and fewer instrumental births (19.3% vs. 30.4%). In our study the small sample size, however, means that these results should be interpreted with caution.

In logistic regression epidural use was independently associated with augmentation of labour. It is generally accepted that epidural anaesthesia can slow the progression of labour [28] leading to the need for augmentation [29]. It could also be that in the presence of augmentation, an epidural is more desirable for women. While there was no meaningful evidence that raspberry leaf use was associated with an increase or reduction in postpartum haemorrhage, augmentation of labour was strongly associated. Postpartum haemorrhage is more common in prolonged labour and in labours that have been augmentation [30].

Fewer neonates had Apgar scores < 7 in the raspberry leaf cohort (9% vs. 15%) and no neonates were admitted to the neonatal intensive care unit. Again, these results are limited by the small sample size and caution must be taken in interpretation. While no adverse outcomes for mothers or babies were observed, this study cannot rule out or provide evidence for frequency of adverse events.

### Limitations

The strength of the association identified in this study between raspberry leaf and augmentation of labour was significantly large. As the effect was so large, our interpretation is that it is not plausible that this was a simple mechanistic effect of an intervention. This suggests the large effect is influenced by some other cause, most likely selection bias. The potential of selection bias has been discussed above. Recruitment methods most likely resulted in a sample that was not representative of the population. While the study design attempted to manage potential confounders both with inclusion criteria (creating a more homogenous sample of low-risk women) and in the analyses, there is potential for unmeasured confounding bias in this study. Furthermore, the small sample size impacts the reliability of findings.

More research is needed to determine the efficacy and safety of raspberry leaf use in pregnancy. A randomised controlled trial (RCT) is needed to provide robust evidence for informed decision making. Given the great discrepancy in the dosage regime, with women having a cup of tea between 1 and 6 times a day and starting between 8 weeks gestation and 38 weeks gestation, these parameters should be included in a future RCT. Potential selection biases may also be minimized in a randomised study where the sample is more representative of the population of interest.

A survey of 121 women in Queensland found that 79% of participants would join a randomised controlled trial on raspberry leaf [1] suggesting that such a study is feasible.

## Conclusion

Noting the limitations of this research, results of this study suggest that raspberry leaf use in pregnancy is associated with a reduction in augmentation of labour, an increase in vaginal birth and no association with postpartum haemorrhage. Many women in Australia are using raspberry leaf in their pregnancy with the aim to improve their birth outcome. While our study demonstrated that raspberry leaf was strongly predictive of women not having their labours medically augmented, the results cannot be relied on or generalised to the wider population of pregnant women. While there were no adverse outcomes recorded in our study, this should not be taken as evidence that raspberry leaf is safe. A randomised controlled trial is urgently needed to provide women and healthcare providers with robust evidence on which to base practice.

### Electronic supplementary material

Below is the link to the electronic supplementary material.

Supplementary Material 1

Supplementary Material 2

## Data Availability

The datasets generated and/or analysed during the current study are not publicly available due to confidentiality of participants. Deidentified datasets are available from the corresponding author on reasonable request.
