# Supplementary material for: Raspberry leaf (Rubus idaeus) use in pregnancy: a prospective observational study
Source: BMC Complement Med Ther. 2024 Apr 22;24:169. doi: 10.1186/s12906-024-04465-7 (PMC11034164; doi:10.1186/s12906-024-04465-7)
Supplement: Supplementary file 1 — Supplementary Material 1 [file 12906_2024_4465_MOESM1_ESM.pdf]

Tools Saved 04/16/2021 Published

Preview Publish

Herbal Medicine in Pregnancy Initial Survey - Consent

ExpertReview score Great

Thank you for taking the time to fill out this survey.

Q15

You are being invited to take part in a research study. Before you decide to take part, it is important for you to understand why the research is being done and what it will involve. Please take time to read the following information carefully by following the link: Participant Information Sheet

Participation in this study is voluntary and involves some short online questionnaires. It is completely up to you whether you participate. If you decide not to participate, it will not affect the treatment you receive now or in the future. Whatever your decision, it will not affect your relationship with the staff caring for you and you can withdraw at any time.

Q11

Herbal Medicine in Pregnancy: Initial Questionnaire

Thank you for agreeing to be part of this research. This questionnaire should only take you less than 5 minutes. Can you please type your first and last name in the text box below. This will remain confidential and will only be used to link your responses to your other questionnaires and birth outcomes for this study.

Q16

Skip to

End of Survey if I understand that taking pa... Is Not Selected

Skip to

End of Survey if I confirm that I have read ... Is Not Selected

Skip to

End of Survey if I understand that I can wit... Is Not Selected

Skip to

End of Survey if I understand that participa... Is Not Selected

Skip to

End of Survey if I give permission for the r... Is Not Selected

Skip to

End of Survey if I understand that my anonym... Is Not Selected

Please mark all the boxes to confirm the following and participate in this study:

- ☐ I confirm that I have read and understood the participant information for this research and I agree to take part in the research
- ☐ I understand that taking part is voluntary and that I am free to withdraw at any time
- ☐ I understand that I can withdraw at any time understand that my data will be stored confidentially and securely in password protected files
- ☐ I understand that participating in this survey will in no way affect the maternity care I recieve
- ☐ I give permission for the researchers to access the clinical data about my pregnancy and birth from hospital records
- ☐ I understand that my anonymity will be protected and I will not be identified in any publications or reports

Q1

What type of maternity care are you accessing for your pregnancy?

- ☐ Private midwife
- ☐ Continuity of Midwife Program eg Canberra Midwifery Program, CaTCH, Calvary Birth Centre
- ☐ Private obstetrician
- ☐ Public hospital
- ☐ GP Shared Care (for example both GP and midwife)
- ☐ Other

Q13

What Hospital is your care with?

- ○ Centenary Hospital for Women & Children
- ○ Calvary Bruce

Q2

Do you take any of the following herbs? Herbal medicine may be in the form of herbal teas, herbal tablets, herbal tinctures (liquid), or herbal powders. You may choose more than one or none. This does not include herbs you eat as food.

- ☐ Peppermint
- ☐ Ginger
- ☐ Raspberry Leaf
- ☐ Chamomile
- ☐ Valerian
- ☐ Evening Primrose Oil
- ☐ Echinacea
- ☐ Cranberry
- ☐ Slippery Elm
- ☐ Other ________
- ☐ I have not taken any herbal medicine

Q4

Are you on an iron supplement?

- ○ Yes
- ○ No

Q14

Display this question

If Are you on an iron supplement? Yes Is Selected

What type of iron do you take?

- ○ Spa Tone
- ○ Floradix
- ○ Ferro-Gradumet
- ○ Ferro-Grad C
- ○ Other ________

Q6

Display this question

If Are you on an iron supplement? Yes Is Selected

How much *elemental* iron (in mg) do you receive in total every day from this supplement? (You may need to read this on the label of your supplement.)

____

Q7

What is your current partnership status?

- ◯ Married
- ◯ Widowed
- ◯ Divorced
- ◯ Separated
- ◯ Never married
- ◯ De facto
- ◯ Single
- ◯ Other

Q8

What did you approximately weigh (in kilograms) before you were pregnant?

Q9

What is your highest level of education?

- ◯ Didn't go to school
- ◯ Year 10 or equivalent
- ◯ Year 12 or equivalent
- ◯ Trade Certificate
- ◯ Associate Diploma
- ◯ Bachelor
- ◯ Postgraduate
- ◯ Other

Q10

What is your total household annual income? This is the combined income that comes into your home each year before tax.

- ◯ Less then $37,000
- ◯ $37,001 - 80,000
- ◯ $80,001 - $180,000
- ◯ Over $180,000

Q11

What was your age at your last birthday?

Q17

Thank you for your time. Another short questionnaire will be emailed to you further along in your pregnancy.
Please type your email address below to receive this.
Make sure you click on the blue arrow at the bottom of the screen when you finish. Thank you.

Import from library

Add new question

Add Block

End of Survey

We thank you for your time spent taking this survey.

Your response has been recorded.
