# Supplementary material for: Raspberry leaf (Rubus idaeus) use in pregnancy: a prospective observational study
Source: BMC Complement Med Ther. 2024 Apr 22;24:169. doi: 10.1186/s12906-024-04465-7 (PMC11034164; doi:10.1186/s12906-024-04465-7)
Supplement: Supplementary file 2 — Supplementary Material 2 [file 12906_2024_4465_MOESM2_ESM.pdf]

Tools Saved Jan 28, 2024 at 4:49 PM Draft Preview Publish

Herbal Use in Pregnancy: Follow up Questionnaire

ExpertReview score Great

Default Question Block

Q1

Herbal Medicine in Pregnancy: Follow up Questionnaire

Thank you once again for being a part of this research and taking the time to fill out this questionnaire. Can you please type your first and last name in the text box below. This will remain confidential and will only be used to link your responses to your other questionnaires and birth outcomes for this study.

Q2

Skip to

End of Survey if No Is Selected

Did you take any herbal medicine in the last 2 weeks? Herbal medicine may be in the form of herbal teas, herbal tablets, herbal tinctures (liquids), or herbal powders. For example slippery elm, ginger, chamomile, peppermint. Please note a 'tisane' is a tea.

- Yes
- No

Q9

Did you take ginger?

- Yes
- No

Q11

Did you take chamomile?

- Yes
- No

Q12

Did you take peppermint?

- ◯ Yes
- ◯ No

Q13

Did you take slippery elm?

- ◯ Yes
- ◯ No

Q14

Did you take evening primrose oil?

- ◯ Yes
- ◯ No

Q15

Skip to

At what week in your pregnancy did yo... if Yes Is Selected

Did you take raspberry leaf?

- ◯ Yes
- ◯ No

Q17

Skip to

End of Survey if Did you take any other herb... Is Not Empty

Did you take any other herbal medicine/s? If so please list here. Please type 'no' if not.

Q10

Skip destination Go to skip origin

At what week in your pregnancy did you start taking raspberry leaf?

Q5

In what form are you taking raspberry leaf? You can choose more than one .

- [ ] Tablet
- [ ] Tea
- [ ] Tincture (liquid)
- [ ] Other - please describe

Q6

If a tea:

Please detail below if you used teabags or leaf, how long you infused in boiling water and how many times per day.

- [ ] How many teaspoons (of leaf) per cup?
- [ ] Teabag
- [ ] Minutes infused for
- [ ] Times per day

Q7

If a tablet:

- [ ] How many milligrams (mgs) in each tablet? (Read label on bottle.)
- [ ] How many tablets a day?

Q18

If a tincture (liquid):

- [ ] How many millilitres (mls) each dose?
- [ ] How many doses a day?

Q19

Did you take any other herbal medicine/s? If so please state.

Q16

Thank you for taking the time to fill out this questionnaire and helping with this research.

Import from library | Add new question

Add Block

End of Survey

We thank you for your time spent taking this survey.

Your response has been recorded.
